# Unearthing the Antibacterial Mechanism of Medicinal Clay: A Geochemical Approach to Combating Antibiotic Resistance

**DOI:** 10.1038/srep19043

**Published:** 2016-01-08

**Authors:** Keith D. Morrison, Rajeev Misra, Lynda B. Williams

**Affiliations:** 1 Biosciences and Biotechnology Division, Physical and Life Sciences Directorate, Lawrence Livermore National Laboratory, Livermore, CA 94550, USA; 2 School of Life Sciences, Arizona State University, Tempe, AZ 85287 USA; 3 School of Earth & Space Exploration, Arizona State University, Tempe, AZ 85287 USA

## Abstract

Natural antibacterial clays, when hydrated and applied topically, kill human pathogens including antibiotic resistant strains proliferating worldwide. Only certain clays are bactericidal; those containing soluble reduced metals and expandable clay minerals that absorb cations, providing a capacity for extended metal release and production of toxic hydroxyl radicals. Here we show the critical antibacterial components are soluble Fe^2+^ and Al^3+^ that synergistically attack multiple cellular systems in pathogens normally growth-limited by Fe supply. This geochemical process is more effective than metal solutions alone and provides an alternative antibacterial strategy to traditional antibiotics. Advanced bioimaging methods and genetic show that Al^3+^ misfolds cell membrane proteins, while Fe^2+^ evokes membrane oxidation and enters the cytoplasm inflicting hydroxyl radical attack on intracellular proteins and DNA. The lethal reaction precipitates Fe^3+^-oxides as biomolecular damage proceeds. Discovery of this bactericidal mechanism demonstrated by natural clays should guide designs of new mineral-based antibacterial agents.

The emergence of antibiotic resistant human pathogens has accelerated inquiries into alternative antibacterial compounds^1^,^2^,^3^,^4^,^5^,^6^. Bacteria rapidly establish resistance against traditional antibiotics that target specific cellular mechanisms DNA replication, protein and cell wall synthesis^2^. As a result, alternative mineral-based therapies against bacterial infections have gained attention^3^,^4^,^5^,^6^,^7^,^8^,^9^. Clays, although used for medicinal purposes throughout millennia, have remained largely unstudied for their applications and reported medical benefits^4^,^5^,^6^. Documented use of reduced metal-rich clays in healing necrotizing fasciitis^6^, commonly known as ‘flesh eating bacteria’, led to our research on the geochemical properties of antibacterial minerals. When tested against a broad spectrum of human pathogens, it was shown that certain clays also kill antibiotic resistant pathogens including methicillin-resistant *Staphylococcus aureus* (MRSA)^6^.

The term ‘clay’ refers to <2 μm minerals of any type, and this size fraction commonly contains discrete clay minerals (smectite, illite, kaolinite), which provide an enormous surface area (100′s m^2^/g) for cation exchange reactions when hydrated^9^. Only a few clays have been identified as antibacterial, completely killing a broad spectrum of human pathogens^6^,^7^,^8^,^10^. Initially we compared the geochemical properties of several antibacterial clays to identify similarities among their mineral assemblages^7^,^8^,^10^. Notably the pH of the hydrated antibacterial clays was either high (>10) or low (<5) where Al and Fe are soluble. This directed our investigation to evaluating the toxicity of soluble elements on pathogenic bacteria.

Antibacterial susceptibility testing of numerous clay deposits led to discovery of a deposit from the Oregon Cascades that is 100% effective at killing all pathogens we have tested so far, including antibiotic resistant strains (see Methods). The antibacterial zones in this deposit formed in hydrothermally altered andesite porphyry associated with argillic clay alteration and volcanogenic massive sulfides^10^. The antibacterial Blue clay zones from this deposit contain mixed layered illite-smectite, pyrite, Ca-plagioclase and quartz. Reconnaissance studies^8^,^10^ showed that this clay kills pathogens by chemical toxicity that occurs in <24 hrs, rather than physical disruption of cells. In nature microbes have evolved in contact with clays, many deriving energy from the minerals^11^,^12^,^13^. However, when antibacterial clays are taken out of their natural environment and hydrated with deionized water for medicinal application they must re-equilibrate with the new fluid. During this process minerals dissolve and oxidize (e.g., pyrite, plagioclase, smectite) releasing metals that stress pathogenic bacteria^10^. Fe, Ca and Al are the major elements released by the Oregon Blue clays when hydrated, therefore we focus on the toxicity caused by Fe and Al, as Ca is not a harmful nutrient for bacteria^14^.

Fe and Al have contrasting roles in biological systems. Fe is essential to nearly all organisms, performing vital cellular functions (e.g., respiration, gene regulation, DNA biosynthesis) as protein cofactors^15^. Oxidized Fe^3+^ is a limiting nutrient for bacterial growth due to its exceedingly low solubility (10^−18^M) at physiological pH, requiring high-affinity siderophore-mediated uptake^15^. In contrast to Fe^3+^, Fe^2+^ is soluble but excess amounts can be toxic to cells, as it increases oxidative stress^16^. Al has no known biological function but is argued to exhibit toxicity through membrane damage^17^. The toxicity of metals is primarily related to their binding affinities to biomolecules in the cell and production of reactive oxygen species (ROS)^1^.

The use of metals as antimicrobial agents is growing in popularity^1^, however antibacterial mechanisms involving soluble metals and minerals have only recently been investigated^3^,^8^,^10^. The aim of this study is to determine how pathogenic bacteria are killed by geochemical reactions that occur during mineral oxidation. We document the antibacterial mechanism of the Oregon Blue clays and provide new insights into metal regulation and toxicity for human pathogens. Using metal toxicity, oxidation and genetic assays, along with advanced bioimaging techniques, we isolate the individual roles of Fe and Al in this natural antibacterial process. Our results have implications for the discovery and synthesis of novel mineral based antibacterial agents.

## Results and Discussion

### Metal Solubility and Production of Reactive Oxygen Species

**Figure 1:**
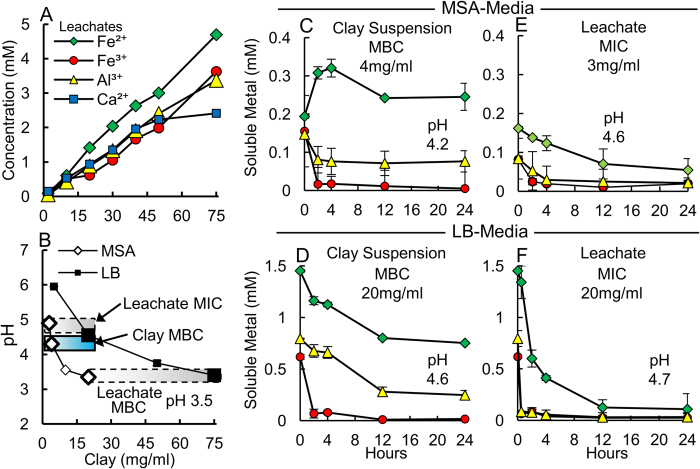
Clay suspensions provide extended metal release whereas aqueous leachates alone precipitate metals. (**A**) Major element concentrations leached from the Blue clays. (**B**) pH, MIC and MBC values for leachates (gray bars) and clay suspensions (blue bar) measured in MSA and LB media. The leachate pH is similar at MBC in both media, while clay suspensions kill bacteria at higher pH. A comparison of soluble metal concentrations (**C,D**) show clay suspensions kill bacteria by maintaining metal solubility over 24 h, while (**E,F**) leachates precipitate metals, only inhibiting growth.

The Oregon Blue clay completely kills a broad range of human bacterial pathogens, including antibiotic resistant strains (see Methods). To understand the antibacterial mechanism exhibited by the Blue clay, metal solubility and production of ROS from clay suspensions in water were measured and compared to metal toxicity from metal solutions alone. Antibacterial susceptibility testing was performed using the model Gram-negative bacterial species *E. coli* (ATCC 25922). The Blue clay mineral assemblage releases mM concentrations of metals (Fe^2+^, Fe^3+^, Al^3+^ and Ca^2+^) when hydrated with deionized water (Fig. 1A, Table S1), through pyrite oxidation, illite-smectite cation exchange and dissolution of plagioclase feldspar^10^. Other metal species (e.g., As, Ag, Hg, Pb, Cu, Zn, Ni) are present in nM to μM concentrations, below levels that inhibit bacterial growth^14^,^18^ (Table S1).

Clay suspensions in deionized water and their aqueous leachates (equilibrated 24 h, the time it takes to kill bacteria) were prepared for antibacterial susceptibly testing. Recognizing that soluble elements speciate differently in various growth media, we compared the Minimum Inhibitory Concentration (MIC) and Minimum Bactericidal Concentration (MBC) of clay suspensions and leachates in two different growth media; minimal salts and amino acids (MSA) media and Lennox broth (LB) (Fig. 1B, Table 1). Increases in pH occurred when clay leachates were mixed with growth media due to metal speciation, causing Fe^3+^ and Al^3+^ precipitation, which reduces their bioavailability and toxicity (Fig. 1C–F). This is an important point because many toxicity studies have not considered precipitation and bioavailability of metals to bacteria^14^,^18^. Speciation calculations (Fig. S2) based on the major inorganic ions in each growth media were performed, organics from the growth media were not included due to the variability of oligopeptides and free amino acids available for reaction^19^. The potential formation of organic ligands with amino acids in the growth media could reduce the concentration of metals available to react with the bacteria. Organic ligands are not thought to contribute to the antibacterial action because the killing occurs at a similar rate in experiments performed with and without growth media^8^. Speciation modeling of clay suspensions at bactericidal pH and Eh conditions show that Fe^2+^ primarily exists in solution as Fe^2+^ and FeSO_4_ in MSA media but in LB precipitates as Fe_3_O_4_. The speciation of Fe^3+^ in both media is dominated by insoluble Fe(OH)_2.7_Cl_0.3_. Aluminum is present as aqueous AlSO_4_^+^ in MSA media but precipitates as Al(OH)_3_ in LB. Calcium is present as aqueous Ca^2+^ and CaSO_4_ in both media. However, these calculations assume that the system is in thermodynamic equilibrium. The antibacterial activity of the Blue clays occurs as the minerals approach a new oxidized equilibrium, with no antibacterial activity occurring in fully oxidized samples^10^. Therefore, thermodynamic speciation calculations will not provide a realistic portrayal of the speciation during the antibacterial mechanism. In order to gauge the concentration of soluble metals available to interact with the bacteria, we monitored the solubility of Fe^2+^, Fe^3+^ and Al^3+^ throughout the antibacterial process. These data are presented in Fig. 1C–F and Tables 1, 2.

Unbuffered clay leachates precipitate metals, and thus require higher concentrations for toxicity (Fig. 1). However, the Blue clay suspensions maintain low pH, while sustaining Fe^2+^ and Al^3+^ release through mineral oxidation and dissolution, requiring a lower dose for toxic effect. Acid and base titrations of the growth media reveal that the LB has a greater buffering capacity compared to the MSA media (Fig. S3). We observed the highest levels of metal precipitation in the LB media due in part to the increased buffering capacity, which maintains a higher pH and precipitates Fe and Al solids (Fig. 1 and S2). The leachate MBC for *E. coli* occurs at pH 3.5, while clay suspension MBC reaches pH 4.2–4.6 (Fig. 1B); indicating that the pH is not the sole factor for killing bacteria. Synthetic metal chloride mixtures of Fe^2+^, Fe^3+^ and Al^3+^, simulating the clay leachate concentrations, produced similar MIC and MBC (Table 1), supporting Fe and Al as the primary bactericidal elements. Importantly, the metal combination is more toxic than single metal solutions (Tables 1, 2).

**Figure 2:**
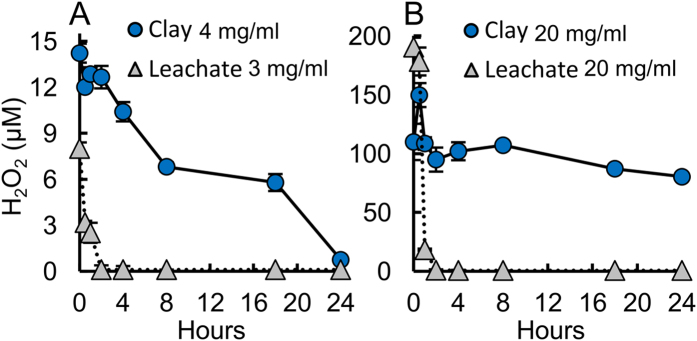
Generation of H_2_O_2_ is maintained by clay suspensions in (**A**) MSA and (**B**) LB-media, while leachates cease production after 2 h. These data show that clay suspensions sustain metal release and H_2_O_2_ production that leads to cell death.

The hydrated Blue clay lowers solution pH < 5, at the MBC, where pyrite oxidation and soluble Fe^2+^ react with molecular oxygen to produce hydrogen peroxide, (H_2_O_2_)^20^ (Fig. 2). Without the clay assemblage, metal oxides precipitate and H_2_O_2_ production stops. The oxidation state of the Blue clay aqueous suspension^10^ reaches Eh ≥ 500 mV. Under these conditions the oxidation of pyrite is known to generate H_2_O_2_ (Fig. 2), which then reacts with Fe^2+^ to form hydroxyl radicals (**·**OH) through the Fenton reaction series^16^,^20^. Clay suspensions produced H_2_O_2_ over 24 h, while aqueous leachates without minerals ceased H_2_O_2_ production after 2 h in both MSA and LB media (Fig. 2A,B), showing that the clay is important as a prolonged source of reactants. Therefore, metal solutions alone are limited as a bactericide due to their inability to sustain H_2_O_2_ production. Pyrite oxidation by molecular O_2_ can directly generate H_2_O_2_ on the crystal surfaces^20^. However, at low pH (<4.5) Fe^3+^ can also oxidize pyrite, releasing soluble Fe^2+^ that can produce H_2_O_2_ 3,20. The expandable smectite component of the Blue clays may serve as a reservoir for Fe^2+^, as divalent cations are preferred over trivalent cations in the smectite interlayer^9^, providing extended Fe^2+^ release when cation exchange occurs^10^ during rehydration for medicinal application. These combined oxidation reactions can explain the elevated H_2_O_2_ levels observed in the Blue clay suspensions, leading to sustained ROS production and killing pathogens via geochemical processes (Fig. 2).

### Metal Hydrolysis

**Figure 3:**
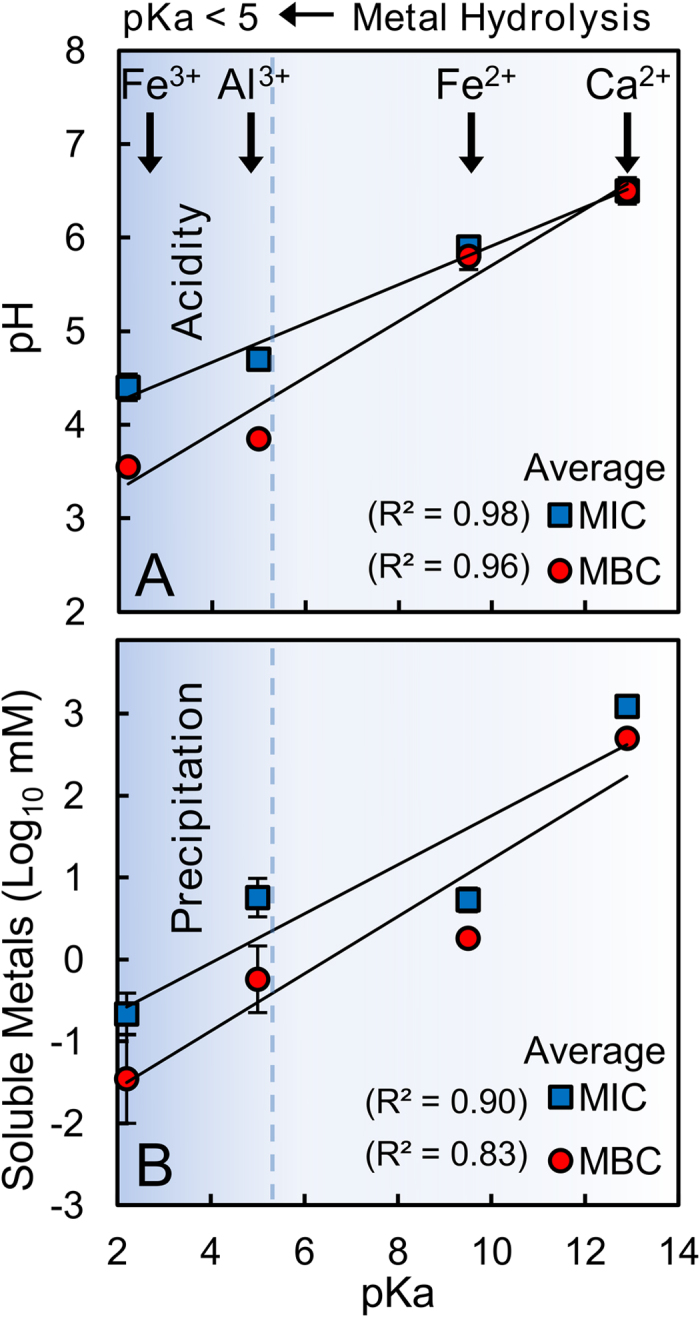
Metal hydrolysis reactions generate acid; therefore, pH, metal solubility and toxicity correlate with pKa. These plots show average MIC and MBC for individual metals in MSA and LB media correlating with (**A**) pH and (**B**) soluble metal content.

Pyrite is present in the Blue clays (3–5%), which can produce sulfuric acid (H_2_SO_4_) during oxidation^10^,^20^. The hydrolysis of soluble metal cations can also generate acid as metals precipitate^21^. The pH and solubility of individual metal cations (Fe^2+^, Fe^3+^ and Al^3+^) were measured at the MIC and MBC in order to evaluate the role of metal hydrolysis reactions relating to metal solubility and toxicity (Table 2). Results show that bacterial metal toxicity is also linked to solution pH and metal solubility, with acidic metal cations (Al^3+^ and Fe^3+^) generating the most acid (Fig. 3). At MIC and MBC, the pH and solubility of individual metals correlate with their pKa (hydrolysis constant). Fe^3+^ and Al^3+^ hydrolysis produce acid that compounds their toxicity (eqn. 1), while precipitation occurs at pH values > pKa^21^. The amount of acid generated through metal hydrolysis is greatest for Fe^3+^ > Al^3+^ > Fe^2+^ > Ca^2+^. Thus, Fe^3+^ alone produced the lowest pH causing cell death at lower concentrations than Fe^2+^ and Al^3+^. Therefore, acid production from Fe^3+^ and Al^3+^ hydrolysis reaction are an overlooked aspect of metal toxicity and may enhance the toxicity of other metals with low pKa values (<5). We observed that leachates and synthetic metal mixtures produced similar pH, indicating that metal hydrolysis plays a greater role in lowering pH than pyrite oxidation in the Oregon Blue clays (Table 1).


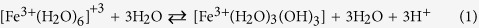


### Bioimaging

**Figure 4:**
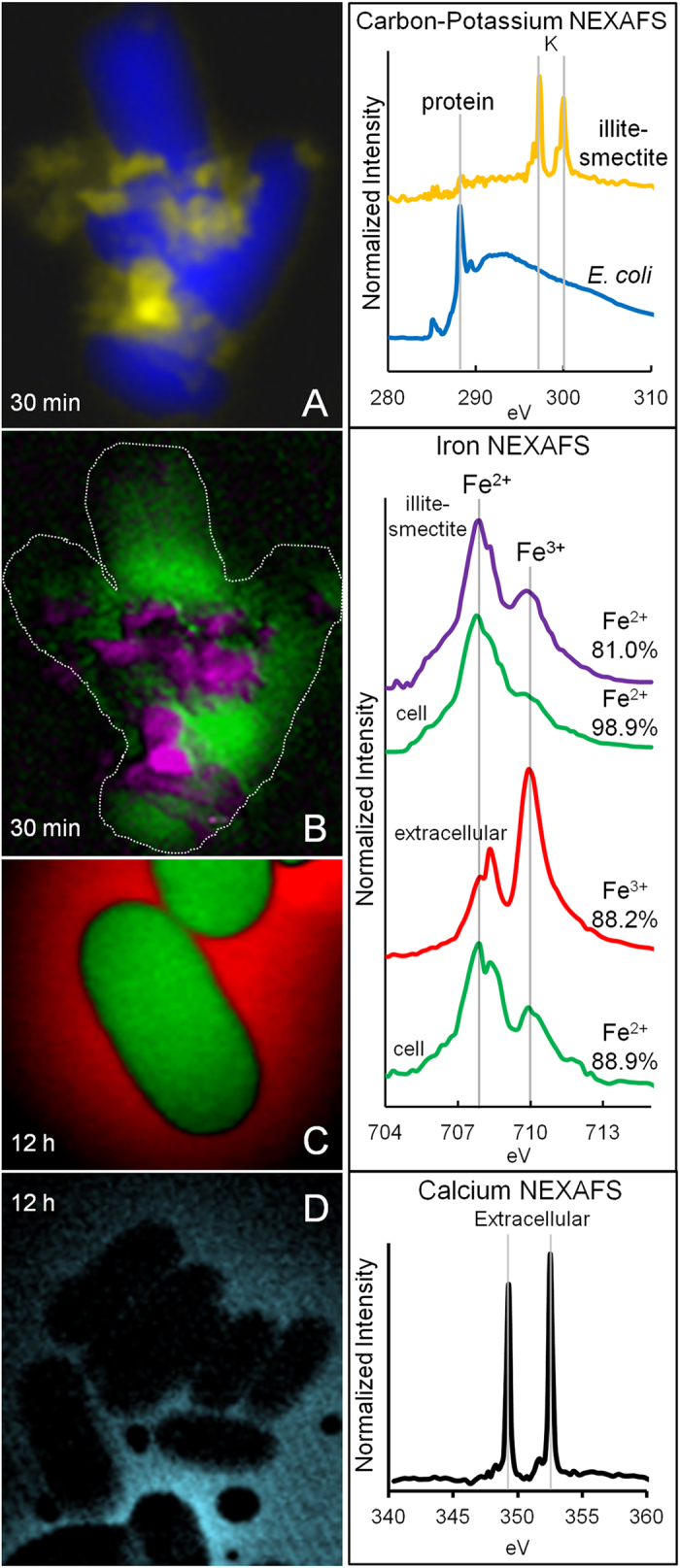
STXM elemental maps of *E. coli* reacted with a clay suspension showing corresponding NEXAFS spectra beside each map (A-D). Elemental maps show (**A**) protein-C from cells (blue) and K (yellow) from illite-smectite (I-S), (**B**) Fe distribution, showing Fe^2+^ adsorption on *E. coli* (green) and Fe in I-S particles (purple), (**C**) *E. coli* cells after 12 h incubation showing preferred Fe^2+^ (green) adsorption, leaving Fe^3+^ (red) in extracellular solution. Percentages of Fe^2+^ and Fe^3+^ were calculated using linear-regression fitting (see supplementary information). (**D**) Ca^2+^ (light blue) remains in solution not on cells. (Variable scales: *E. coli* width = 0.5 μm).

**Figure 5:**
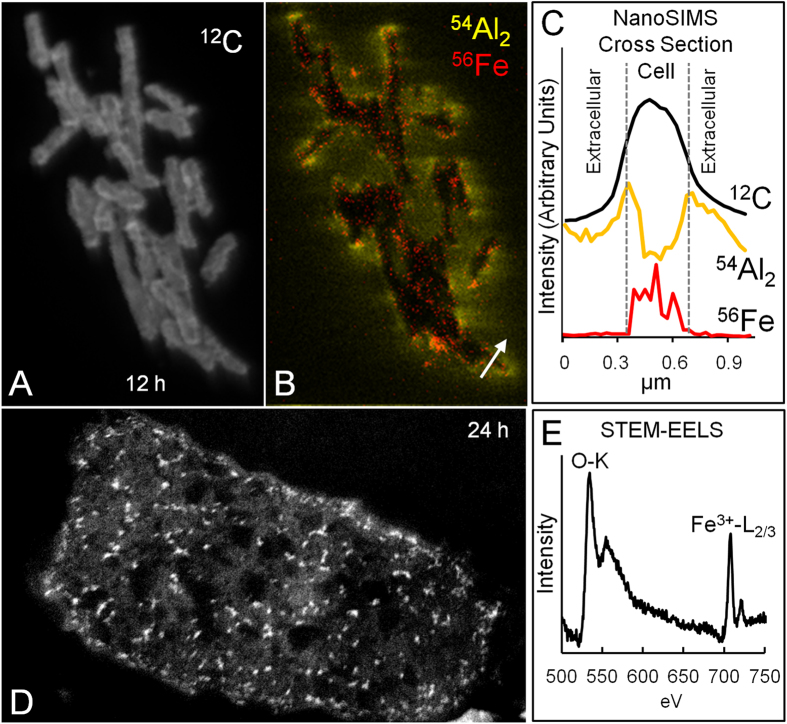
NanoSIMS image of *E.coli* showing, (**A**) ^12^C map of cells, (**B**) ^54^Al_2_ outside (yellow) and ^56^Fe (red) inside cells. (**C**) A cross section of ion counts across the white arrow in (**B**). (**D**) STEM image of *E. coli* showing intracellular nanoparticles (white spots) developed after 24 h incubation. (**E**) EELS spectra of nanoparticles showing O (K-edge) and Fe^3+^ (L_2/3_-edge) identifying white spots as Fe^3+^-oxide precipitates. (Scale: *E. coli* width = 0.5 μm).

Bacteria adsorb a range of metals on their cell envelopes due to a high anionic charge density from phosphate and carboxyl groups^22^,^23^. We used elemental bioimaging to examine the adsorption, location and redox state of metals reacting with *E. coli* (Figs 4 and 5). Clay suspensions (100 mg/ml) reacted with *E. coli* were analyzed in hydrated state using synchrotron scanning transmission X-ray microscopy (STXM, Advanced Light Source 11.0.2) for C, K^+^, Ca^2+^, Fe^2+^ and Fe^3+^ (Fig. 4A–D). Near edge x-ray absorption fine structure (NEXAFS) spectra of C and K show K-rich particles adhered to cells (Fig. 4A). Illite-smectite (I-S) dominates the deposit mineralogy^10^ and is the likely source of K. Linear-regression fitting of Fe spectra with reference compounds^24^ (Fig. S1) shows that Fe is present in I-S particles predominantly as Fe^2+^ (81%). Most likely this Fe^2+^ is located in the I-S octahedral sites or interlayers (Fig. 4B). Soluble Fe^2+^ (89–99%) is adsorbed preferentially to cell envelopes (Fig. 4B,C), while Ca^2+^ remains in solution (Fig. 4D). In *E. coli*, Ca^2+^ and Mg^2+^ adsorbed to phosphate-rich lipopolysaccharides provide outer membrane stability; however, under acidic conditions H^+^ can displace these cations^25^.

Nano-scale secondary ion mass spectrometry (NanoSIMS) maps of C, Al and Fe were generated to determine their binding sites in the cell (Fig. 5A,B). Cells were measured after reaction with a 100 mg/ml Blue clay suspension for 12 hrs. A clump of *E. coli* cells were sputtered with the primary ion beam, exposing the cell interior and Fe and Al maps were generated (see Methods). NanoSIMS maps of Al and Fe (Fig. 5B,C) show that Al binds to bacterial membranes, while Fe enters the cell. *E. coli* that were not reacted with Blue clay showed no intracellular Fe (below detection limit), consistent with previous studies indicating 354 and 2662 ppm intracellular Fe concentrations in control and clay reacted cells, respectively^8^. The mechanism for Al toxicity is unknown, but it has been shown to compete with Ca and Mg for phosphate binding and may damage membranes^17^. Previous measurements of extracellular elements in *E. coli* reacted with the Oregon Blue clay leachates, indicated that Fe and Al replace membrane bound Ca and Mg^8^. STEM bioimaging of *E. coli* treated with Blue clay leachates show intracellular nanoparticles (electron dense white spots in Fig. 5D) after 24 hr incubation, coincident with cell death. Using scanning transmission electron microscopy-electron energy loss spectra (STEM-EELS) these spots were determined to be Fe^3+^-oxides (Fig. 5D,E).

The STXM and NanoSIMS bioimaging results reveal that bacterial membranes remain enriched in Fe^2+^ and Al^3+^ throughout the antibacterial process (Figs 4 and 5). The Blue clay suspensions maintain H_2_O_2_ generation over 24 hrs (Fig. 2), which leads to production of toxic hydroxyl radicals (**·**OH) upon reaction with membrane adsorbed Fe^2+^ and encapsulates the cell in an oxidizing environment. The STEM-EELS images (Fig. 5D) showing Fe^3+^-oxide accumulations inside the cells likely formed from the oxidation of excess intracellular Fe^2+^ which may enter the cell through low affinity uptake systems^14^. The production of intracellular Fe^3+^-oxides requires oxidation, accompanied by formation of **·**OH. Therefore, below we argue that the Fe^3+^ oxides reflect the transition to an oxidizing environment inside the cell, where proteins and DNA are damaged.

### Protein Oxidation

Hydroxyl radicals are generated as Fe^2+^ is oxidized by H_2_O_2_ through the Fenton reaction^16^,^26^. The proximity of **·**OH generation to biomolecular targets is crucial to its toxicity because this radical exists only briefly (10^–9^ s half-lives) and diffuses only nanometers before reacting^26^. Thus, Fe^2+^-enriched bacterial membranes (Fig. 4C), reacting with H_2_O_2_ formed outside the cells (Fig. 2), will generate **·**OH in direct proximity to membrane proteins and lipids. Therefore, intracellular Fe^2+^ is required to generate **·**OH that reacts with intracellular biomolecules, precipitating Fe^3+^-oxides where oxidative stress occurs (Fig. 5D).

**Figure 6:**
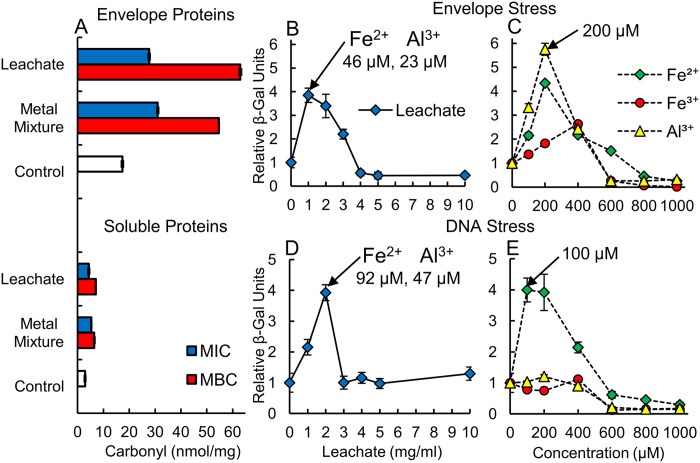
Clay leachates produce high levels of envelope protein oxidation, activating σ^E^ and SOS stress responses. Plots show (**A**) protein carbonyl content (nmol-carbonyl/mg-protein) measured in envelope vs. soluble protein fractions of *E. coli* reacted with leachates and metal mixtures in MSA media, at MIC and MBC. (**B**) Envelope stress measured by σ^E^-response from LacZ fusions in leachates compared to (**C**) single metal solutions. (**D**) DNA stress (SOS-response) measured on *E. coli* reacted with leachates, and (**E**) single metal solutions. LacZ results are normalized to levels measured in control *E. coli* (Relative β-Gal Units).

We evaluated protein oxidation by measuring carbonyl content^27^ in separated membrane and soluble protein fractions of *E. coli* reacted with clay leachates and metal solutions (Fig. 6A). Membrane fractions of *E. coli* showed that the greatest protein oxidation (30–60 nmol-carbonyl/mg-protein) occurred upon exposure to leachates and metal solutions, while the soluble protein fractions (dominated by intracellular proteins) had lower carbonyl content (4–7 nmol/mg). This indicates that bacteria exposed to antibacterial clays first experience ROS stress targeting membranes, followed by intracellular protein oxidation in response to the influx of Fe^2+^.

### Genetic Responses

Our results show that the synergistic activities of Fe^2+^, Fe^3+^ and Al^3+^ produce greater toxicity to bacteria at lower concentrations than individual metals. LacZ reporter gene fusions were employed to quantify bacterial stress responses to clay leachates and metals^28^,^29^,^30^. Genetic responses to bacterial envelope and DNA stress were evaluated in MSA-media using *rpoHP3*::*lacZ* (σ^E^-response) and *sulA*::*lacZ* (SOS-response) gene fusion constructs (Fig. 6B–E). Outer membrane protein (OMP) misfolding from ROS can activate the σ^E^-response, transcribing genes whose products regulate OMP proteolysis and folding^28^,^29^. Leachates caused the highest activation of σ^E^, increasing *rpoHP3*::*lacZ* expression 4-fold upon reaction with a 1 mg/ml leachate solution (Fig. 6B,C). Weaker envelope stress occurred for individual Fe^2+^ and Al^3+^ solutions, requiring 77% more metal to reach stress levels similar to the clay leachate. This further supports the synergistic role of Al^3+^ and Fe^2+^ for membrane damage. Misfolding of OMPs by Al^3+^ may aid in protein oxidation by exposing amino acids to **·**OH attack, supporting Al^3+^ as a pro-oxidant^31^. Pathogenic Gram-negative bacteria activate the σ^E^-response to protect against ROS generated by host macrophages^27^. Our results demonstrate that metal binding to bacterial envelopes is capable of misfolding proteins, followed by ROS oxidation. Consequently, antibacterial clays may act in a manner similar to macrophages by engulfing and killing bacteria with metal-based ROS attack.

Intracellular oxidative stress can cause single strand breaks in DNA, triggering the SOS-response in *E. coli*, suspending cell growth until DNA is repaired^30^. Leachates and Fe^2+^ solutions produced similar DNA stress (Fig. 6D,E), while Fe^3+^ and Al^3+^ alone were not genotoxic. Thus, intracellular stress results from excess Fe^2+^ uptake. *E. coli* cells take in Fe^2+^ through high and low affinity uptake systems (Feo, ZupT), while trivalent cations (Fe^3+^) must enter through high affinity siderophore systems (TonB-ABC transporters)^15^. Al^3+^ substitution in Fe-S proteins, resulting in free intracellular Fe and ROS stress, has been proposed as a mechanism of Al^3+^ toxicity in Gram-negative bacteria^32^. However, our bioimaging (Fig. 5B,C) and genetic data show that Al^3+^ does not enter the cell or trigger the SOS-response. The toxicity of Al to cell membranes has been noted by several studies of acid rain^17^,^33^. In particular Williams^17^ (1999) found that Al^3+^ can only be transported into cells by chelators, as there are no pumps or channels for free Al^3+^ uptake or rejection. Because Al^3+^ cannot be reduced it accumulates at cell membranes where it bonds strongly to small oxygen donor ligands, such as phosphates^17^,^33^. Other researchers^34^,^35^ have observed that Al^3+^ alone does not produce lipid peroxidation, but enhances the Fe^2+^ peroxidation of phospholipids. Our research shows the combination of Fe^2+^ and Al^3+^ produces a greater genetic response to protein misfolding and oxidation in the outer membrane of *E. coli*, while genotoxicity only arises from the uptake and intracellular oxidation of Fe^2+^ (Fig. 6).

## Conclusion

**Figure 7:**
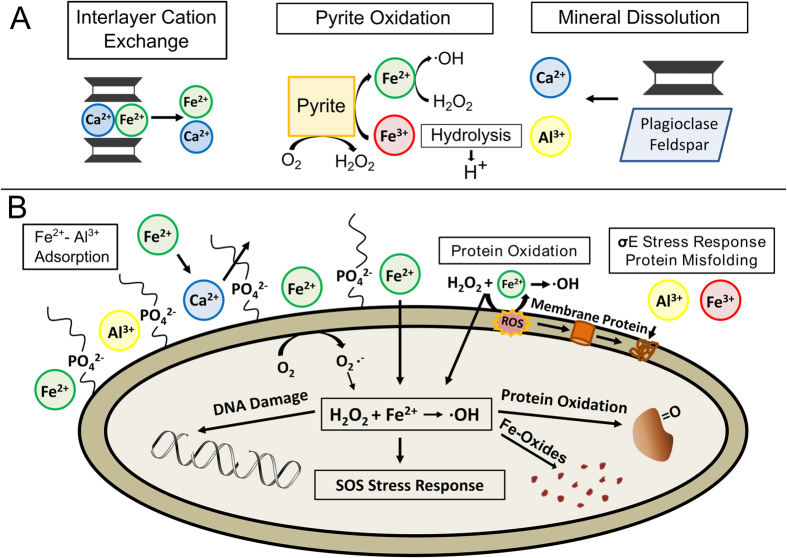
Schematic showing the antibacterial mechanism of the Oregon Blue clays. (**A**) Illite-smectite interlayer cation exchange, pyrite oxidation and mineral dissolution (plagioclase feldspar and I-S) provide soluble Fe^2+^, Fe^3+^, Al^3+^ and Ca^2+^, while generating H_2_O_2_. Hydrolysis and precipitation of Fe^3+^ and Al^3+^ sustain the acidic environment. (**B**) Fe^2+^ and Al^3+^ out compete Ca^2+^ binding to phosphate rich lipopolysaccharides on the outer membrane of *E. coli*, which results in protein misfolding and oxidation, activating the σ^E^-stress response. Hydrogen peroxide generated extracellularly by clay suspensions diffuses through the cell envelope and reacts with intracellular Fe^2+^, forming radicals that oxidize proteins and DNA, activating the SOS-response. Fe^3+^-oxide precipitates coincident with cell death. Through extended metal release and H_2_O_2_ production, the Oregon blue clays simultaneously stress multiple cellular systems unlike traditional antibiotics.

The essential components for the antibacterial activity of the Oregon Blue clay are Fe^2+^, Fe ^3+^ and Al^3+^, which work synergistically to overcome the highly evolved metabolic functions of human pathogens. The hydrated antibacterial clays generate a low pH (<4.6) environment, through mineral oxidation, dissolution and hydrolysis reactions, sustaining metal release and ROS production throughout the antibacterial mechanism (Fig. 7A). Expandable smectite interlayers provide a reservoir for Ca^2+^ and Fe^2+^ exchange. Pyrite oxidation by dissolved O_2_ generates H_2_O_2_ on the mineral surfaces, while oxidation by Fe^3+^ releases Fe^2+^ into solution. Hydroxyl radicals are generated as Fe^2+^ is oxidized to Fe^3+^ by H_2_O_2_, providing additional Fe^3+^ for pyrite oxidation. Mineral dissolution (plagioclase and I-S) releases Al^3+^ and Ca^2+^ into solution. Hydrolysis of Fe^3+^ and Al^3+^ generate H^+^, maintaining low pH (<4.6), promoting mineral dissolution and metal solubility (Fig. 7A).

The reactions outlined above generate Fe^2+^ and Al^3+^ that synergistically attack pathogenic bacteria in an oxidizing, metal rich environment that damages multiple cellular components (Fig. 7B). Fe^2+^ and Al^3+^ both accumulate on cell envelopes, impairing membranes through protein misfolding (Al^3+^) and oxidation (Fe^2+^), activating the σ^E^-stress response. Cell envelopes remain enriched in Fe^2+^, providing a constant source of **·**OH attack on the cell envelope. Fe^2+^, not Al^3+^, floods the cytoplasm generating intracellular **·**OH that damages DNA and proteins, marking oxidation sites with Fe^3+^-oxide precipitates (Fig. 7B). This constant intracellular oxidation causes single strand DNA breaks and overwhelms the cells defense mechanisms against ROS. The critical components identified in this natural antibacterial process should guide designs for new mineral-based antibacterial agents.

## Materials and Methods

### Antimicrobial Susceptibility Testing

The broad-spectrum antibacterial activity of the Oregon Blue clay was determined by testing against pathogenic bacteria: *Escherichia coli* (ATCC 25922), extended-spectrum β-lactamase (ESBL, β-lactam resistant) *E. coli* (ATCC 51446), *Salmonella enterica* serovar Typhimurium (ATCC 14028), *Pseudomonas aeruginosa* (ATCC 27853), *Staphylococcus epidermidis* (ATCC 14990), methicillin-resistant *S. epidermidis* (MRSE) (ATCC 35948), *Staphylococcus aureus* (ATCC 29213), methicillin-resistant *S. aureus* (MRSA) Sonora Quest Laboratories (Tempe AZ, USA). Bacteria were grown in Lennox Broth (LB), then diluted in fresh media to a concentration of ~10^7^ cells per ml. The Blue clay was sterilized by autoclaving at 121 °C and 15psi for 1h to kill environmental microbes. Next, 100 mg of the sterilized clay was added to 200 μl of the diluted culture and incubated at 37 °C for 24 h with constant rotary shaking. Cell death was measured by serial dilution and plating on LB-agar and incubation at 37 °C for 24 h^36^. All bacterial strains listed above were killed 100%.

*E. coli* (ATCC 25922) MIC and MBC toxicity measurements were performed in Lennox broth (LB) and minimal salts and amino acids (MSA) to evaluate effects of minimal and rich media on pH, metal solubility, and toxicity. The MSA media, modified from^37^, contained 1 g (NH_4_)_2_SO_4_, 0.06 g MgSO_4_·7 H_2_O, 0.06 g CaCl_2_, 0.02 g KH_2_PO_4_, 0.03 g Na_2_HPO_4_, 2.383g 4-(2-hydroxyethyl)-1-piperazineethanesulfonic acid (HEPES), 1 ml FeSO_4_ (10 mM), 1% casein amino acids and 0.4% glucose per liter. The rich media consisted of 20 g per liter LB and 0.4% glucose per liter. Overnight cultures of *E. coli* grown in 20 g per liter LB were diluted in MSA or LB media (1:50, 5 × 10^7^ cells) and incubated at 37 °C for 30 min. Aliquots (0.5 ml) of mineral leachates (see next section), metal solutions (Fe^2+^, Fe^3+^, Al^3+^ and Ca^2+^) or metal mixtures of Fe^2+^: Fe^3+^: Al^3+^ (1.0: 0.6: 0.7 ratio) simulating the clay leachate proportions were then mixed with *E. coli* cultures (0.5 ml) in a 48 well plate (1:1 ratio of media: metal). The synthetic metal solutions and mixtures were diluted using freshly prepared 100 mM stock solutions of FeCl_2_, FeCl_3_, AlCl_3_ and CaCl_2_ • 2H_2_O. The well plates were then incubated at 37 °C on a rocker plate for 24h.

Metal precipitation can prevent the accurate measurement of MIC values using visible spectroscopy Optical Density (OD) at 600 nm. A method to remove excess metal precipitates without cell lysis was performed using an oxalic acid-EDTA mixture^38^. The oxalic acid-EDTA (Ox-EDTA) mixture consisted of 1.86 g EDTA-Na_2_-H_2_O, 1.47 g sodium citrate, 0.074 g KCl, 0.5 g NaCl and 1.26 g oxalic acid adjusted to pH 8 with 10 M NaOH. After 24 h of incubation with leachates or metals, 0.5 ml of culture was added to 0.5 ml of the Ox-EDTA reagent and reacted for 30 min. at room temperature. Cells were then pelleted and re-suspended in 1 ml Ox-EDTA reagent, reacted for 30 min., pelleted and re-suspended in 0.5 ml isotonic NaCl (0.9%). Metal rinsed cells were then transferred into a 98 well microplate and cell growth was measured at 600 nm with a microplate reader. MBC concentrations were determined by plating 50 μl of culture in triplicate on LB-agar plates after 24 h incubation at 37 °C.

### Mineral Leachates

Mineral leachates were prepared by ultra-sonicating samples with deionized water (1 min.), followed by wrist action shaking for 24 h to chemically equilibrate for the amount of time poultices are applied, and then centrifuged (15,000 rpm, 1 h) to separate minerals from the leachate solution. Elemental analyses of mineral leachates and metal solutions were performed using a Thermo ICAP-Quadrupole ICP-MS using a multi-element internal standard in combination with external calibration standards which were analyzed at the beginning of each measurement session. X-Series quadrupole ICP-MS. Samples were acidified in 2% nitric acid prior to analysis.

Mineral leachates and metal solutions were measured for Fe^2+^, Fe^3+^ and total iron^10^. For the measurement of Fe^2+^ a 50–500 μL aliquot of leachate was added to 500 μl of 2.5 wt% 1, 10 phenanthroline (dissolved in 95% ethanol). The solutions were then diluted with 1 wt% sodium citrate to 1.5 ml. Absorbance was measured with a spectrophotometer at 510 nm after 15 minutes for color development. Measurement of Fe^3+^ was achieved in the same manner using 100 μL of 10 wt% hydroxylamine hydrochloride as a reducing reagent. Stock solutions of ferrous ammonium sulfate hexahydrate and ferric chloride (200 mg/L) were prepared and acidified using 3.6N sulfuric acid to produce standard curves ranging from 1–8 mg Fe/L.

### Metal Solubility

The effect of bacterial growth media on metal precipitation was investigated to determine the actual soluble metal content compared to the amount added in the first hour of incubation. Mineral leachates or metal solutions were reacted with MSA or LB media in a 1:1 ratio and incubated for 30 min. at 37 °C (or 24 h for time series). Samples were then transferred into 1.5 ml centrifuge tubes and centrifuged for 30 min. at 13,000 rpm. Supernatant solutions were diluted with 6M nitric acid (1:2) to prevent metal precipitation before analysis. Each growth media was digested in 6M nitric acid overnight and analyzed for Fe and Al. The concentrations of Fe and Al (≤5 μM) in the growth were added to the metal concentrations reported for the MIC and MBC measurements in order to evaluate the total metal concentration interacting with the bacteria. Metal content was measured using ICP-MS after dilution in 2% nitric acid.

### Speciation Modeling

Speciation diagrams for Fe, Al, Ca, S, P and Cl were calculated using the HYDRA/MEDUSA program^39^. Speciation calculations were performed using initial elemental concentrations from mineral suspensions at MBC in MSA (4 mg/ml, clay) and LB (20 mg/ml, clay). In the MSA media, concentrations of Fe^2+^, Fe^3+^, Al^3+^ and Ca^2+^ were set at 0.2, 0.15, 0.15 and 0.2 mM, respectively equivalent to the bactericidal concentrations for each element. The major anions (SO_4_^2−^, PO_4_^3−^ and Cl^−^) were set at 4, 0.2 and 0.3 mM, respectively. Concentrations of Fe^2+^, Fe^3+^, Al^3+^ and Ca^2+^ in LB media were set at 1.4, 1.0, 0.8 and 1.0 mM, respectively based on their measured MBC. Anion concentrations in LB for SO_4_^2−^ and Cl^−^ were set at 4, and 21 mM, respectively. Williams *et al.*, 2011 identified SO_4_^2−^ as the major leachate anion, with SO_4_^2−^ concentrations (mM) ~2 times greater than Fe. Eh values were set at 500 mV for both media and mole fractions were calculated from pH 1 to 7.

### pH and Mineral Titrations

All pH and mineral titrations were measured using an Orion Dual Star pH meter (LE409 pH electrodes). Media titrations were measured using 10 ml of either LB or MSA media mixed with 10 ml de-ionized water instead of leachate. The media was continually stirred and titrated with 1 × 10^−3^ N HCl or NaOH in 1 ml increments to a total of 70 mL using a burette. The pH meter was allowed to stabilize 1–5 minutes between each acid or base addition.

### Hydrogen Peroxide Assay

H_2_O_2_ levels were measured for mineral leachates and mineral suspensions reacted with *E. coli* growing in LB or MSA media using a method modified from Cohen^40^. Aliquots were sampled over a 24 h period and centrifuged at 13,000 rpm for 2 min. Supernatants (500–20 μl) were transferred into micro centrifuge tubes with the addition of 50 mM EDTA. Buffer solution (1M KH_2_PO_4_, pH 4.2) was added to a final volume of 1400 μl. Then 50 μl of 5 mM leuco crystal violet was added followed by 50 μl of horseradish peroxidase type II (14.4 units/ml). Absorbance was measured with a spectrophotometer at 590 nm wavelength after 15 min for color development. Hydrogen peroxide standard curves ranging from 0.5–25 μM were prepared in the same manner.

### Scanning Transmission X-ray Microscopy (STXM)

STXM images were measured at the Advanced Light Source (Berkeley, CA) using the 11.0.2 beamline. Data processing was performed using the aXis2000 software package (http://unicorn.mcmaster.ca). A 100 mg/ml mineral suspension was shaken for 24 h and allowed to flocculate 30 min. forming a dilute mineral suspension. An overnight culture of 2 ml *E. coli* grown in LB (20 g/l) was pelleted, re-suspended in the dilute mineral suspension (2 ml) and incubated on an orbital shaker. Aliquots (0.5 μl) were sampled and pipetted onto a silicon nitride window (Si_3_N_4_ 75 nm thick). A second Si_3_N_4_ window was placed on top of the first window, sealed with epoxy and placed into the STXM sample chamber and purged with helium. Scans of Fe, C, K and Ca were measured using a 1ms dwell time and 0.2eV steps to avoid radiation damage^41^. Transmitted signals were converted to optical density by obtaining the incident flux through a portion of the Si_3_N_4_ cell with no bacteria. Elemental component maps were generated by identifying target spectra using cluster analysis followed by principal component analysis to identify the spectral features in the image. Reference spectra^24^ for Fe^2+^ Cl_2_ and Fe^3+^ Cl_3_ were used for quantitative analysis of each target spectra. The energy scale (eV) for Fe^2+^-and Fe^3+^-rich target spectra was normalized to the Fe reference compounds (Fe^2+^–707.8 and Fe^3+^–709.8). Spectral intensities were normalized to the Fe reference spectra and linear regression stack fitting was performed to quantitatively measure Fe^2+^ and Fe^3+^ levels^24^.

### Nano Secondary Ion Mass Spectrometry (NanoSIMS)

*E. coli* cells, reacted as described above, were sampled after 12 h and pelleted by centrifugation. Cells were then fixed in 2.5% glutaraldehyde for 2 h, re-pelleted, dehydrated in 50%, 75% and 100% ethanol then dried onto silicon wafers. Isotopic imaging was done using a Cameca (Ametek) NanoSIMS 50L with a 16keV Cs^+^ primary ion beam at ~2pA current (~50 nm probe size using 300 μm D1-2 apertures). This beam was rastered over a 12 μm^2^ region containing *E. coli* cells with a dwell time of 10msec/pixel (256^2 pixels) for 8 layers (1.5 h measurement). Secondary negative ions of ^12^C^–^, ^27^Al_2_^–^, and ^56^Fe^–^ were collected simultaneously using electron multiplier detectors. Mass resolution was set to separate isobaric interferences of ^54^Fe^–^ from ^27^Al_2_^–^ (which makes more negative ions than ^27^Al^–^) and ^40^Ca^16^O^–^ from ^56^Fe^–^ and was achieved using a 220 μm entrance slit.

### Scanning Transmission Electron Microscopy- Electron Energy Loss Spectroscopy

#### (STEM-EELS)

Bacterial-mineral suspensions in (as described above) were imaged using STEM-EELS to determine the elemental composition and redox state of intracellular precipitates. Aliquots of the bacterial-mineral suspension were pelleted after 24 h and fixed using a BalTec HPM010 high-pressure freezer and freeze substitution using 2% OsO_4_, embedded in Spurr’s resin, sectioned to 60 nm and mounted on gold TEM grids^10^. STEM-EELS images were collected using a JEOL 2010F STEM at 200 kV and a resolution of 0.5 eV to image the O-K edge and the Fe-L_2,3_ edges.

### Protein Carbonylation

*E. coli* overnight cultures (5 ml) grown in LB (20 g/l) were pelleted by centrifugation at 3,000 rpm for 10 min. Pellets were then resuspended in 2.5 ml MSA media and reacted with 2.5 ml of mineral leachate or metal mixture and incubated at 37 °C for 24 h. Samples were pelleted and re-suspended in 5 ml lyse buffer (1 mM EDTA, 10mM TRIS Buffer) 3 times and placed on ice. DNase I and PMSF (0.1 mM) were added prior to cell lyses using a French pressure cell. Lysed cells were then centrifuged at 3,000 rpm for 10 min. to remove cell debris. Membrane and soluble protein fractions were separated by ultra-centrifugation under vacuum (40,000 rpm, 4 °C) for 1 h. Nucleic acids were removed from the soluble protein fraction (supernatant) using a nucleic acid removal kit (ProFoldin, NAR911). Soluble proteins were transferred into centrifuge tubes and sodium dodecyl sulfate (SDS) was added to a concentration of 10%. Membrane fractions were resuspended in 10% SDS and placed in a 42 °C water bath for 10 min. Splits of each protein fraction were reacted with 10 mM dinitrophenyl hydrazine (DNPH) in 2.5N HCl (carbonyl content) or 2.5N HCl (control) at room temperature for 30 min. Proteins were pelleted using 10% ice cold trichloroacetic acid and centrifuged at 13,000 rpm for 30 min. in a cold room (4 °C). Proteins were then re-suspended in ethanol-ethylacetate (1:1) for 30 min. and pelleted (x3) to remove excess DNPH. Protein pellets were dried and re-suspended in 6M guanidine hydrochloride and absorbance was measured at 370 nm (subtracting control absorbance). Carbonyl content was calculated using the molar extinction coefficient for DNPH (22,000 M^−1^ cm^−1^). Protein content was determined using the Bradford protein assay kit (Pierce Coomassie Protein Assay^TM^) and calibrated with bovine serum albumin. Results are expressed as nmol-carbonyl/mg protein (nmol carbonyl/mg)^27^.

### Genotoxicity and envelope stress

Determination of bacterial stress responses to envelope damage and genotoxicity were carried out using *sulA*::*lacZ* (SOS-response) and *rpoHP3*::*lacZ* (σE-response) LacZ fusions, respectively. β-galactosidase activity was measured using 4-methylumbelliferyl β-D-galactopyranoside (MUG) fluorescence^42^. Mineral leachates, metals and metal mixtures were reacted in MSA media (1:1 ratio) for 3 h (37 °C) at 1–10 mg/ml and 100–1000 μM concentrations, respectively. Samples were removed from the incubator and placed on ice for 10 min. The MSA media did not result in visible metal precipitation at leachate and metal concentrations less than 10 mg/ml and 1000 μM, respectively. Cell growth was measured (OD600) after transferring 200 μl of culture to a 96 well plate. Then 200 μl of culture was transferred into 800 μl of freshly prepared Z-buffer. Next 250 μl of MUG (1 mg/ml) was added and incubated at room temperature for exactly 30 min. upon which 300 μl of NaCO_3_ (1M) was added to stop the reaction. Fluorescence was measured (Cary eclipse fluorescence spectrophotometer) using an excitation wavelength of 360 nm and an emission wavelength of 455 nm. Arbitrary β-gal units were calculated using the following equation below, which normalizes the MUG fluorescence at 460 nm to incubation time (*t*) and cell density A (OD 600):


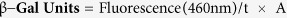


## Additional Information

**How to cite this article**: Morrison, K. D. *et al.* Unearthing the Antibacterial Mechanism of Medicinal Clay: A Geochemical Approach to Combating Antibiotic Resistance. *Sci. Rep.* **6**, 19043; doi: 10.1038/srep19043 (2016).

## Supplementary Material

Supplementary Information

## Figures and Tables

**Table 1:** MIC, MBC and pH values for Blue clay leachates and aqueous metal mixtures (Fe^2+^, Fe^3+^, Al^3+^) reacted with *E. coli* growing in MSA and LB media for 24 hr at 37 °C.

Leachate	MIC/MBC	pH	MSA-Media											
			Initial (mM)			Soluble (mM)			% Soluble					
			Fe^2+^	Fe^3+^	Al^3+^	Fe^2+^	Fe^3+^	Al^3+^	Fe^2+^	Fe^3+^	Al^3+^			
3 mg/ml	MIC	4.9	0.162	0.082	0.084	0.137	0.016	0.038	84.6	19.5	45.2			
20 mg/ml	MBC	3.4	1.45	0.62	0.79	1.42	0.074	0.65	97.9	11.9	82.3			
Metal Mixture	MIC	5.1	0.160	0.063	0.087	0.124	BDL	0.023	77.5	0.0	26.4			
Metal Mixture	MBC	3.9	1.10	0.55	0.78	0.93	BDL	0.43	84.5	0.0	55.1			
			**LB-Media**											
20 mg/ml	MIC	4.7	1.45	0.62	0.79	1.34	0.012	0.084	92.4	1.9	10.6			
75 mg/ml	MBC	3.5	4.70	3.63	3.05	2.23	0.063	1.74	47.4	1.7	57.0			
Metal Mixture	MIC	5.0	1.92	1.25	1.38	1.60	0.055	0.11	83.3	4.4	8.0			
Metal Mixture	MBC	3.6	3.91	2.62	3.22	3.82	0.155	0.67	97.6	5.9	20.8			

pH was measured after addition of leachates or metal mixtures to bacteria in media. Initial metal content is reported, along with soluble metal content measured in growth media after 1 h.

Note: relative standard deviation for pH and elemental analysis were <1.0% and <10.0%, respectively.

**Table 2:** Individual metal MIC, MBC and pH values for *E. coli* measured in MSA and LB media.

Cation	MIC/MBC	MSA-Media				LB-Media			
		pH	Initial(mM)	Soluble(mM)	% Soluble	pH	Initial(mM)	Soluble(mM)	% Soluble
**Fe^2+^**	MIC	5.9	2	1.7	85.0	5.9	5	1.8	36.0
	MBC	5.6	8	7.4	92.5	5.7	8	3.8	47.5
**Fe^3+^**	MIC	4.3	0.5	0.01	2.0	4.5	3	0.15	5.0
	MBC	3.5	1	0.12	12.0	3.6	5	0.39	7.8
**Al^3+^**	MIC	4.7	0.5	0.22	44.0	4.7	3	1.5	50.0
	MBC	3.9	7	3.3	47.1	3.8	11	9.8	89.1
**Ca^2+^**	MIC	6.4	500	–	–	6.6	500	–	–
	MBC	6.1	1000	–	–	6.4	1500	–	–

pH was measured directly after addition of metals to bacteria in media. Initial metal content was determined along with soluble metal content in media alone after 1 h.

Note: relative standard deviations for pH were <1%; elemental analysis <8.0%.

## References

[b1] Lemire J. A., Harrison J. J. & Turner R. J. Antimicrobial activity of metals: mechanisms, molecular targets and applications. Nat Rev Microbiol 11, 371–384 (2013). 23669886 10.1038/nrmicro3028

[b2] Walsh C. Molecular mechanisms that confer antibacterial drug resistance. Nature 406, 775–781 (2000). 10963607 10.1038/35021219

[b3] Friedlander L. R., Puri N., Schoonen A. A. & Karzai W. The effect of pyrite on *Escherichia coli* in water: proof-of-concept for the elimination of waterborne bacteria by reactive minerals. J Water Health 13.1, 42–53 (2015). 25719464 10.2166/wh.2014.013 PMC5891221

[b4] Carretaro M. I. Clay minerals and their beneficial effects upon human health. A review. Appl Clay Sci 21, 155–163 (2002).

[b5] Ferrell R. E. Medicinal clay and spiritual healing. Clays Clay Miner 56, 751–760 (2008).

[b6] Williams L. B., Holland M., Eberl D. D., Brunet T. & Brunet de Courrsou L. Killer clays! Natural antibacterial clay minerals. Miner Soc Bull 139, 3–8 (2004).

[b7] Williams L. B., Haydel R. F., Giese R. F. & Eberl D. D. Chemical and mineralogical characteristics of French green clays used for healing. Clays Clay Miner 56, 437–452 (2008). 19079803 10.1346/CCMN.2008.0560405 PMC2600539

[b8] Williams. L. B. *et al.* What Makes a Natural Clay Antibacterial? Environ Sci Technol 45, 3768–3773 (2011). 21413758 10.1021/es1040688 PMC3126108

[b9] Moore D. M. & Reynolds R. C. In X-ray Diffraction and the Identification and Analysis of Clay Minerals 2^nd^ edition. (Oxford University Press, 1997).

[b10] Morrison K. D., Underwood J. C., Metge D. W., Eberl D. D. & Williams L. B. Mineralogical variables that control the antibacterial effectiveness of a natural clay deposit. Environ Geochem Health 36, 613–631 (2014). 24258612 10.1007/s10653-013-9585-0

[b11] Kostka J. E., Dalton D. D., Skelton H., Sherry D. & Stucki J. W. Growth of iron(III)-reducing bacteria on clay minerals as the sole electron acceptor and comparison of growth yields on a variety of oxidized iron forms. Appl Environ Microbio 68, 6256–6262 (2002). 10.1128/AEM.68.12.6256-6262.2002 PMC134413 12450850

[b12] Church C. D., Wilkin R. T., Alpers C. N., Rye R. O. & McCleskey R. B. Microbial sulfate reduction and metal attenuation in pH 4 acidic mine water. Geochemical Transictions 8, 1–14 (2007). 10.1186/1467-4866-8-10 PMC2211471 17956615

[b13] Bhowal S. & Chakraborty. R. Five novel acid-tolerant oligotrophic thiosulfate-metabolizing chemolithotrophic acid mine drainage strains affiliated with the genus *Burkholderia* of *Betaproteobacteria* and identification of two novel soxB gene homologues. Res Microbiol 162, 436–445 (2011). 21349327 10.1016/j.resmic.2011.02.007

[b14] Nies D. H. Microbial heavy-metal resistance. Appl Microbiol Biotechnol 51, 730–750 (1999). 10422221 10.1007/s002530051457

[b15] Andrews S. C., Robinson A. K. & Rodríguez-Quiñones F. Bacterial iron homeostasis. FEMS Microbiol Rev 27, 215–237 (2003). 12829269 10.1016/S0168-6445(03)00055-X

[b16] Imlay J. A., Chin S. M. & Linn S. Toxic DNA damage by hydrogen peroxide through the Fenton reaction *in vivo* and *in vitro*. Science 240, 640–642 (2008). 2834821 10.1126/science.2834821

[b17] Williams R. P. What is wrong with aluminium? J Inorg Biochem 76, 81–88 (1999). 10612060 10.1016/s0162-0134(99)00118-x

[b18] Harrison J. J., Turner R. J. & Ceri H. High-throughput metal susceptibility testing of microbial biofilms. BMC Microbiol 5, 1471–2180 (2005). 10.1186/1471-2180-5-53 PMC1262724 16202124

[b19] Sezonov G., Joseleau-Petit D. & D’Ari R. *Escherichia coli* physiology in luria-bertani broth. J Bacteriol 189, 8746–8749 (2007). 17905994 10.1128/JB.01368-07 PMC2168924

[b20] Schoonen M. A., Harrington A. D., Laffers R. & Strongin D. R. Role of hydrogen peroxide and hydroxyl radical in pyrite oxidation by molecular oxygen. Geochim Cosmochim Acta 74, 4971–4987 (2010).

[b21] Pearson R. G. Acids and bases. Science 151, 172–177 (1966). 17746330 10.1126/science.151.3707.172

[b22] Beveridge T. J. & Koval F. Binding of metals to cell envelopes of *Escherichia coli* K-12. Appl Environ Microbiol 42, 325–335 (1981). 7025758 10.1128/aem.42.2.325-335.1981 PMC244009

[b23] Fein J. B., Daughney C. J., Yee N. & Davis T. A. A chemical equilibrium model for metal adsorption onto bacterial surfaces. Geochim Cosmochim Acta 61, 3319–3328 (1997).

[b24] Dynes J. J. *et al.* Speciation and quantitative mapping of metal species in microbial biofilms using scanning transmission X-ray microscopy. Environ Sci Technol 40, 1556–1565 (2006). 16568770 10.1021/es0513638

[b25] Borrok D. *et al.* The effect of acidic solutions and growth conditions on the adsorptive properties of bacterial surfaces. Chem Geol 209, 107–119 (2004).

[b26] Winterbourn C. C. Reconciling the chemistry and biology of reactive oxygen species. Nat Chem Biol 4, 278–286 (2008). 18421291 10.1038/nchembio.85

[b27] Levine R. L. *et al.* Determination of carbonyl content in oxidatively modified proteins. Method Enzymol 186, 464–478 (1990). 10.1016/0076-6879(90)86141-h 1978225

[b28] Alba B. M. & Gross C. A. Regulation of the *Escherichia Coli* σ^E^ dependent envelope stress response. Mol Microbiol 52, 613–619 (2004). 15101969 10.1111/j.1365-2958.2003.03982.x

[b29] Raivio T. L. Envelope stress responses and Gram-negative bacterial pathogenesis. Mol Microbiol 56, 1119–1128 (2005). 15882407 10.1111/j.1365-2958.2005.04625.x

[b30] Huisman O., D’Ari D. & Gottesman S. Cell-division control in Escherichia coli: specific induction of the SOS function SfiA protein is sufficient to block septation. Proc Natl Acad Sci 81, 4490–4494 (1984). 6087326 10.1073/pnas.81.14.4490 PMC345616

[b31] Exley C. The pro-oxidant activity of aluminum. Free Radical Bio Med 36, 380–387 (2004). 15036357 10.1016/j.freeradbiomed.2003.11.017

[b32] Lemire J., Mailloux R., Auger C., Whalen D. & Appanna V. D. Pseudomonas fluorescens orchestrates a fine metabolic-balancing act to counter aluminium toxicity. Env Microbiol 12, 1384–1390 (2010). 20353438 10.1111/j.1462-2920.2010.02200.x

[b33] Garcidueñas Piña R. & Cervantes C. Microbial interactions with aluminum. BioMetals 9, 311–316 (1996). 8696081 10.1007/BF00817932

[b34] Aruoma O. I., Halliwell B., Laughton M. J., Quinlan G. J. & Gutteridge J. M. C. The mechanism of initiation of lipid peroxidation. Evidence against a requirement for an iron(II)-iron(III) complex. Biochem J 258, 617–620 (1989). 2706005 10.1042/bj2580617 PMC1138407

[b35] Ohyashiki T., Karino T., Suzuki S. & Matsui K. Effect of aluminum ion on Fe^2+^-induced lipid peroxidation in phospholipid liposomes under acidic conditions. J. Biochem. 120, 895–900 (1996). 8982853 10.1093/oxfordjournals.jbchem.a021503

[b36] National Committee for Clinical Laboratory Standards. Methods for Dilution Antimicrobial Susceptibility Tests for Bacteria that Grow Aerobically: Approved Standard. NCCLS Document M7-A5 (ISNB 1-56238- 394-9) fifth ed. NCCLS, Pennsylvania, USA (2000).

[b37] Teitzel G. M. & Parsek M. R. Heavy metal resistance of biofilm and planktonic *Pseudomonas aeruginosa*. Appl Environ Microbiol 69, 2313–2320 (2003). 12676715 10.1128/AEM.69.4.2313-2320.2003 PMC154819

[b38] Tovar-Sanchez A., Saundo-Wilhelmy S. A., Garcia-Vargas M., Weaver R. S., Popels L. C. & Hutchins D. A. A trace metal clean reagent to remove surface bound iron from marine phytoplankton. Mar Chem 82, 91–99 (2003).

[b39] Puigdomenech I. Hydra/Medusa Chemical Equilibrium Database and Plotting Software. Stockholm: KTH – Royal Institute of Technology (2004).

[b40] Cohen C. A., Pak A., Strongin D. & Schoonen M. A. Quantifying hydrogen peroxide in iron containing solutions using leuco crystal violet. Geochem Trans 6, 47–51 (2005). 10.1186/1467-4866-6-47 PMC1475790 35412761

[b41] Warwick T. *et al.* A scanning transmission x-ray microscope for materials science spectromicroscopy at the advanced light source. Rev Sci Instrum 69, 2964–2973 (1998).

[b42] Vidal-Aroca F. *et al.* One-step high-throughput assay for quantitative detection of β-galactosidase activity in intact Gram-negative bacteria, yeast, and mammalian cells. Bio Tech 40, 433–440 (2006). 10.2144/000112145 16629389

