# Supplementary material for: Unearthing the Antibacterial Mechanism of Medicinal Clay: A Geochemical Approach to Combating Antibiotic Resistance
Source: Sci Rep. 2016 Jan 8;6:19043. doi: 10.1038/srep19043 (PMC4705759; doi:10.1038/srep19043)
Supplement: Supplementary Information [file srep19043-s1.pdf]

Supplementary Information for:

Unearthing the Antibacterial Mechanism of Medicinal Clay: A Geochemical Approach to Combating Antibiotic Resistance

Keith D. Morrison[1*], Rajeev Misra[2], Lynda B. Williams[1]

[1] School of Earth & Space Exploration, Arizona State University, Tempe, AZ 85287 USA
[2]School of Life Sciences, Arizona State University, Tempe, AZ 85287 USA

*Corresponding Author:
Keith D. Morrison
School of Earth & Space Exploration
Arizona State University
Tempe, AZ 85287 USA
Phone: 951-314-7113
Keith.Morrison@asu.edu

Supplementary Figures

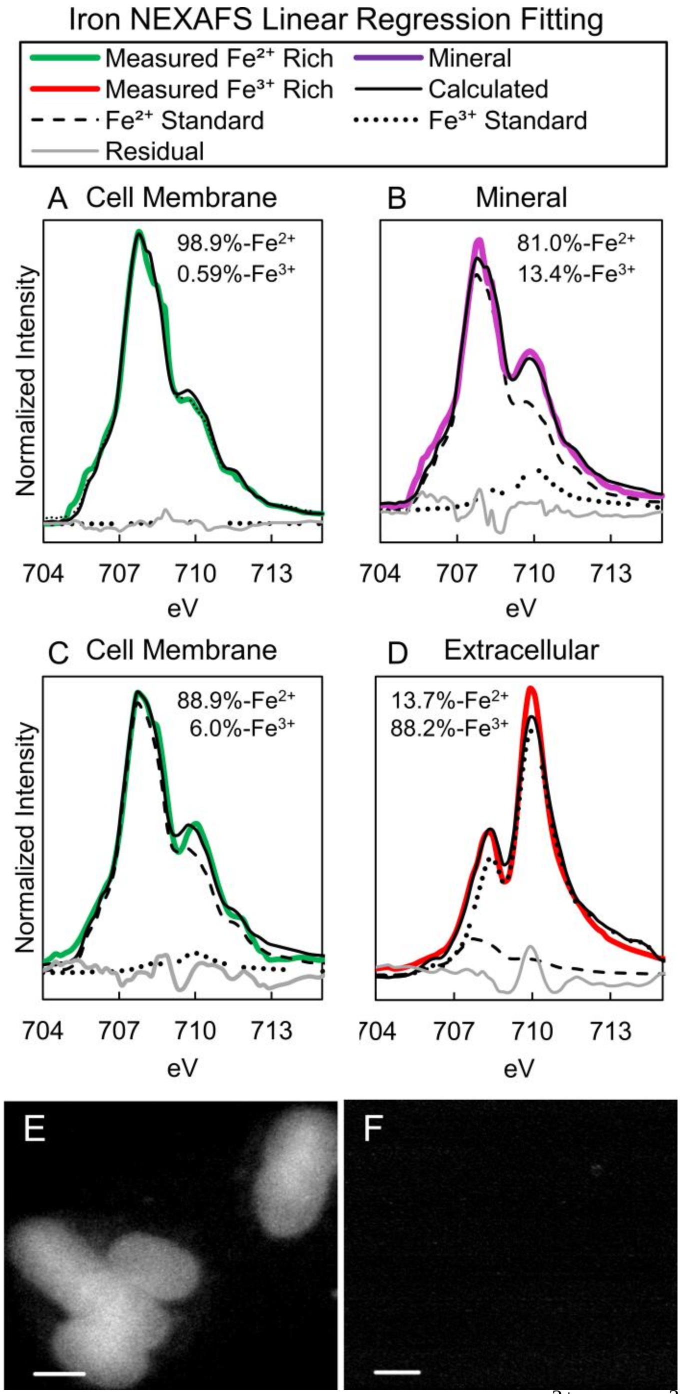

Figure S1. STXM linear regression fitting of Fe-L_3 edge using Fe^{2+} and Fe^{3+} reference compounds[24]. *E. coli* cells reacted with clay suspensions after 30min. showing Fe percentages associated with (A) cell membranes and (B) mineral particles (Fig. 3B). (B) *E. coli* cell membrane Fe percentages and (D) extracellular percentages after 12h (Fig. 3C). (E) STXM protein maps (280-288.2 eV) of *E. coli* cells reacted with a 100mg/ml mineral suspension (12h) and washed with Ox-EDTA to remove adsorbed metals[37]. (F) Fe maps (700-708 eV) of the same cells showing no signal. Scale bars represent 0.5µm.

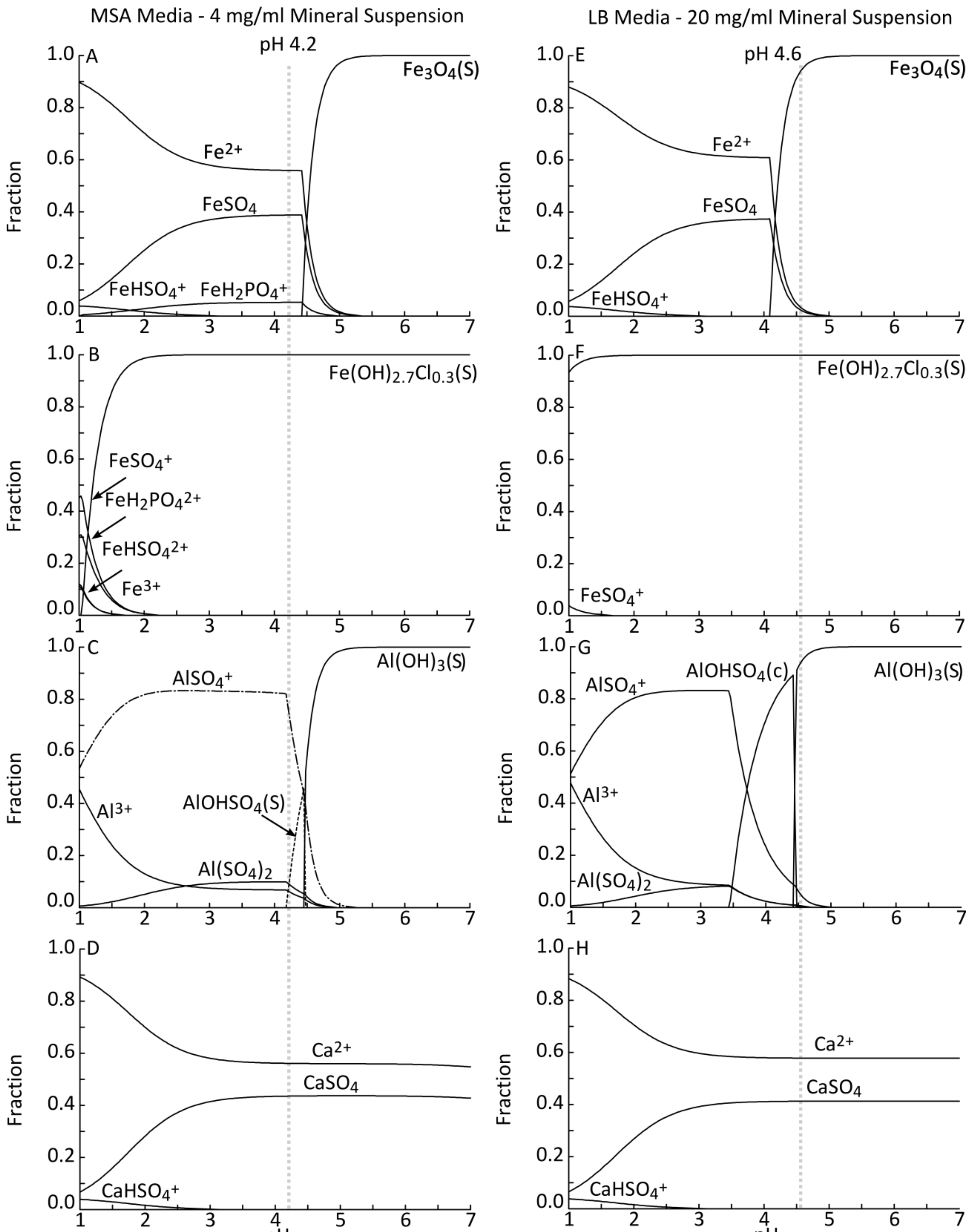
MSA Media - 4 mg/ml Mineral Suspension
LB Media - 20 mg/ml Mineral Suspension
pH 4.2
pH 4.6
A
$Fe_3O_4(S)$
Fe^{2+}
$FeSO_4$
$FeHSO_4^+$
$FeH_2PO_4^+$
B
$Fe(OH)_{2.7}Cl_{0.3}(S)$
$FeSO_4^+$
$FeH_2PO_4^{2+}$
$FeHSO_4^{2+}$
Fe^{3+}
C
$Al(OH)_3(S)$
$AlSO_4^+$
Al^{3+}
$AlOHSO_4(S)$
$Al(SO_4)_2$
D
Ca^{2+}
$CaSO_4$
$CaHSO_4^+$
E
F
G
$AlOHSO_4(c)$
H
Fraction
pH

Figure S2.

Figure S2. Speciation diagrams for Fe, Al, Ca, S, P and Cl were calculated using initial elemental concentrations from mineral suspensions at MBC in MSA (4 mg/ml, clay) and LB (20 mg/ml, clay) media. (A-D) Speciation in MSA media with concentrations of Fe^{2+}, Fe^{3+}, Al^{3+} and Ca^{2+} set at 0.2, 0.15, 0.15 and 0.2 mM, respectively, equivalent to the bactericidal concentrations leached form the clay. The major anions in MSA (SO_4^{2-}, PO_4^{3-} and Cl^-) were set at 4, 0.2 and 0.3 mM, respectively. (E-H) Concentrations of Fe^{2+}, Fe^{3+}, Al^{3+} and Ca^{2+} in LB media were set at 1.4, 1.0, 0.8 and 1.0 mM, respectively, based on their MBC measurements. Anion concentrations in LB for SO_4^{2-} and Cl^- were set at 4, and 21 mM, respectively. Eh values were set at 500 mV for both media and mole fractions were calculated from pH 1 to 7 using the HYDRA/MEDUSA program[39].

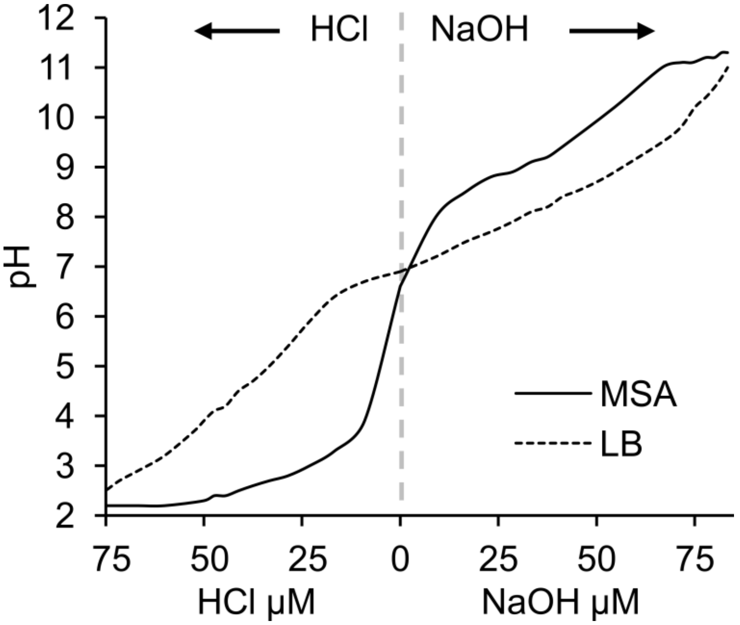

Figure S3. Acid and base titrations of MSA and LB growth media. Changes in pH were monitored during the addition of 0.1 mM HCl or NaOH in 1 ml aliquots.

Supplementary Table

Table S1. Elemental analyses of clay leachates at various concentrations. All values are reported at micro-molar (μM) concentrations. Elements <1nM are below detection limit (BDL).

Element	3 mg/ml	20 mg/ml	75 mg/ml	100 mg/ml
**Na**	6.7	7.7	23.6	34.2
**Mg**	22.2	137.7	485.8	749.2
**Al**	83.7	794.8	3046.3	4998.0
**P**	0.8	12.4	122.6	233.3
**K**	1.9	1.2	0.3	0.7
**Ca**	113.3	733.1	2413.4	3933.7
**Ti**	2.4E-03	1.0E-02	0.1	0.2
**V**	4.8E-03	0.2	0.8	1.3
**Cr**	1.5E-02	0.1	0.5	0.8
**Mn**	0.8	5.5	19.5	30.0
**Fe^{2+}**	161.7	1452.1	4693.4	5885.2
**Fe^{3+}**	81.6	618.0	3625.3	5456.3
**Co**	0.2	1.2	4.2	6.5
**Ni**	0.2	1.6	5.4	8.3
**Cu**	0.4	2.2	7.6	11.8
**Zn**	0.2	1.0	3.7	5.8
**As**	3.8E-03	0.1	0.7	1.4
**Se**	1.7E-02	0.2	0.5	0.8
**Rb**	1.1E-02	1.9E-02	0.1	3.8E-02
**Sr**	0.6	3.8	11.9	18.0
**Zr**	1.3E-03	1.7E-03	3.3E-03	5.0E-03
**Mo**	5.6E-03	2.0E-02	0.1	0.1
**Cd**	BDL	2.2E-03	5.5E-03	8.0E-03
**Cs**	4.7E-03	9.1E-03	2.6E-02	2.1E-02
**Ba**	0.1	2.9E-02	2.0E-02	1.6E-02
**Hf**	BDL	BDL	BDL	BDL
**W**	3.7E-03	2.0E-03	1.9E-03	1.6E-03
**Ag**	BDL	BDL	BDL	BDL
**Hg**	BDL	BDL	BDL	BDL
**Pb**	BDL	BDL	BDL	BDL
**U**	BDL	BDL	1.5E-03	2.3E-03

Note: relative standard deviations were <7.0 %
